# Correlation between sports visual ability and catching performance in elite collegiate baseball infielders: a position-specific analysis

**DOI:** 10.3389/fphys.2026.1828207

**Published:** 2026-05-25

**Authors:** Yuankun Long, Zhao Zhang, Dexin Wang, Qiang Wei, Youlin Xiao, Chao Chen, Xinbo Zhang

**Affiliations:** 1 College of Physical Education, Dalian University, Dalian, China; 2 College of Physical Education, Xi ‘an University of Architecture and Technology, Xi ‘an, China; 3 School of Athletic Performance, Shanghai University of Sport, Shanghai, China

**Keywords:** catching accuracy, cross-validation, eye-hand coordination, Senaptec assessment, stereoscopic vision

## Abstract

**Objective:**

To explore the relationship between sports visual ability and specific performance of 24 elite collegiate baseball infielders (including first basemen, second basemen, third basemen, and shortstops) from the Dalian University School of Physical Education.

**Methods:**

This study employed a cross-sectional correlational design to examine the relationship between sports visual abilities and catching performance among 24 infielders from the baseball team of the Dalian University School of Physical Education. The sports visual ability indices of 24 infielders from the baseball team of the Dalian University School of Physical Education were evaluated using the Senaptec system. The specific performance was reflected by the correct catch rate (success rate for 50 random pitches). Multiple linear stepwise regression analysis was used to analyze the predictive utility of visual abilities for correlation between the two.

**Results:**

Key findings revealed that depth perception (stereoscopic vision) demonstrated the strongest negative association with catching performance (R = -0.844, P < 0.001), followed by eye-hand coordination average reaction time and perceptual span (R = -0.729 and R = 0.800, respectively; all P < 0.001). Additional visual abilities, including near/far quickness, target capture, and multi-object tracking, also exhibited moderate to strong associations with performance (absolute R values ranging from 0.467 to 0.573; all P < 0.001). Multiple linear stepwise regression identified depth perception, eye-hand coordination metrics, Go/No go total score, and perceptual span as significant predictors, collectively predicting 93.2% of the variance in catching performance (adjusted R² = 0.932, P < 0.001). A leave-one-out cross-validation (LOOCV) was conducted to assess model generalizability, yielding a cross-validated R² (Q²) of 0.87, which confirms that the model is not severely over-fitted despite the modest sample size.

**Conclusion:**

In this cross-sectional study of 24 collegiate baseball infielders, depth perception, perceptual span, eye-hand coordination, reaction time, near/far quickness, and target capture were found to be significantly associated with laboratory-based catching performance. These findings suggest that these visual abilities may be important correlates of catching accuracy in this controlled setting; however, their direct causal role in on-field performance enhancement requires further investigation through longitudinal and ecologically valid studies.

## 1 Introduction

Baseball is a sport that demands extremely high levels of skill and accuracy, requiring athletes to possess outstanding physical qualities such as speed, agility, and strength (Egan and Zierath, 2023; Hoffman et al., 2009). It is widely believed that baseball players possess superior visual acuity compared to the general population, with baseball often regarded as the sport with the highest visual demands (Laby et al., 2018). Numerous studies have shown that professional and collegiate baseball infielders exhibit better visual acuity, contrast sensitivity, and visual tracking abilities than the average person (Filippini et al., 2025). Elite baseball infielders demonstrate superior dynamic visual acuity, faster oculomotor responses, and stronger sensorimotor performance (Ho et al., 2023). Specifically, infielders must process multidimensional visual information simultaneously in highly dynamic environments, including pitch trajectory and baserunner displacements. Their eye-hand coordination and stereo visual acuity are significantly higher than those of other positions (Laby et al., 2018). Therefore, 24 baseball infielders (6 first basemen, 6 second basemen, 6 third basemen, and 6 shortstops) were recruited for this study.

Despite these recognized demands, previous research has primarily focused on visual reaction time (Hülsdünker et al., 2018), dynamic visual acuity (Ho et al., 2023), binocular coordination (Laby et al., 2018), and decision-making (Toole et al., 2021; Liu et al., 2020). However, existing research predominantly focuses on static visual assessments, while dynamic visual competency frameworks and performance prediction models for infielders remain unexplored, resulting in training protocols that lack position-specificity (Tremblay et al., 2025). Moreover, inconsistencies in assessment methods have led to conflicting conclusions across studies. Prior research on batters used static visual acuity tests (Laby et al., 2018), whereas studies on pitchers focused on retinal function (Klemish et al., 2018). Critically, most studies overlooked position-specific demands: infielders require simultaneous processing of pitch trajectory, baserunner movements, and glove positioning—skills not captured by conventional assessments. Research on associations between sports visual abilities and infielder-specific performance remains scarce.

To address this gap, this study employed the Senaptec system, whose reliability and validity have been validated in baseball (Klemish et al., 2018; Liu et al., 2020), ice hockey (Poltavski and Biberdorf, 2015), and softball (Appelbaum and Erickson, 2018). We evaluated 10 categories of sports visual ability indicators in 24 collegiate baseball infielders and examined their associations with specialized fielding and catching performance. By identifying key visual predictors and exploring latent visual-motor constructs, this study aims to provide ecologically valid, position-specific insights to inform tailored visual training programs for infielders.

## 2 Materials and methods

### 2.1 Materials

The study selected a total of 24 male athletes from the baseball team of Dalian University (Liu et al, 2020). The study included 24 male collegiate baseball infielders from the Dalian University team. Because this was a position-specific exploratory study conducted within a single elite collegiate team, the sample size was determined by the number of all eligible infielders who met the inclusion criteria during the study period. This recruitment strategy was chosen to maximize representativeness within this specialized population. In addition, the sample size was informed by previous sports-vision studies using comparable experimental designs and similarly specialized athlete populations (e.g., Liu et al., 2020; Klemish et al., 2018). We therefore considered the present sample appropriate for an exploratory analysis of visual-performance associations in this cohort, while acknowledging that larger multi-center samples are needed to confirm the stability and generalizability of the regression findings. Furthermore, a *post-hoc* power analysis was conducted using G*Power 3.1. Based on the observed effect sizes (R² = 0.932 for the regression model), our sample achieved a statistical power (1-β) > 0.95 for detecting large effects at α = 0.05, indicating that the sample was sufficient to reliably identify the strong relationships central to this investigation. Their average age was 18.30 ± 4.10 years, and their average training experience was 4.41 ± 1.83 years. The study selected a total of 24 male infielders from the baseball team of Dalian University, comprising 6 first basemen, 6 second basemen, 6 third basemen, and 6 shortstops. This position-specific sampling was implemented to ensure alignment between the standardized catching task (18 m distance, random pitches) and the defensive demands routinely faced by infielders, who must rapidly judge ground balls or line drives in a spatial range of approximately 15–20 m from home plate. All athletes possessed technical qualifications at National Level 2 or higher, a criterion commonly used to define “elite” or “highly trained” status in Chinese collegiate sports contexts (Wang and Sulaiman, 2025), ensuring a sample of athletes with homogeneous competitive experience and skill proficiency. Inclusion criteria comprised current enrollment as a competitive baseball player at Dalian University, normal or corrected-to-normal visual acuity, no history of severe ocular pathology or surgery, and ongoing participation in formal collegiate baseball competitions. Exclusion criteria included visual impairments not correctable to normal, recent concussions, or musculoskeletal injuries affecting performance.

The participants followed the unified training schedule of the Dalian University baseball team, training no fewer than 5 days per week, with each session lasting at least 2 hours. The testing protocol and procedures were approved and supported by the team’s coaching staff prior to implementation. Meanwhile, this study protocol was approved by Bureau of the National Institute of Sports Science of China (Approval Number: CISS2024.09.02). Additionally, all participants were thoroughly informed about the testing content and signed informed consent forms, ensuring compliance with research regulations and safeguarding the rights of the participants.

### 2.2 Methods

#### 2.2.1 Sports visual ability testing

1. Timing of Testing: Based on the training and competition schedule of the Dalian University baseball team, and to avoid conflicts with matches that could affect testing progress, the sports visual ability test was scheduled for November 13, 2024, after discussions with the coaching staff. The sports visual ability test and the catching performance test were conducted one day apart.
2. Testing Indicators: The Senaptec system was used to classify and explain the testing indicators (**Table 1**) (Erickson et al., 2011). A total of 10 indicators were assessed: Visual Clarity (VC), Contrast Sensitivity (CS), Depth Perception (DP), Near/Far Quickness (N/FQ), Perceptual Span (PS), Multiple Object Tracking (MOT), Reaction Time (RT), Target Capture (TC), Eye-Hand Coordination (EHC), and Decision-Making (Go/No-Go, GNG) (Mann et al., 2007). Each test is carried out under standardized conditions in accordance with the manufacturer’s guidelines.
3. Testing Methods: The Senaptec (USA) system was evaluated in accordance with the fixed sequence specified by the system (**Table 1**), following its standard protocol and based on the test design of Jones et al. (2016) and Liu et al. (2020). Before the test, participants received clear oral guidance and completed exercises for each task to ensure understanding. Short rest intervals are provided between each test indicator to minimize fatigue. All tests were conducted in a controlled laboratory environment with consistent ambient lighting and viewing distances.

**Table 1:** Detailed description of sports vision ability testing.

Test indicator	Methodology	Evaluation criteria
VC (Visual Clarity	Participants hold a mobile device and stand 3 meters from the tablet. They identify the gap direction of C-shaped patterns displayed on the tablet screen and swipe the corresponding direction on the mobile device. Testing order: monocular (left/right eye) followed by binocular.	Higher accuracy correlates with smaller identifiable patterns. Unit: logMAR (lower values indicate better performance). Scored using a 5-point system (5 = highest score).
CS(Contrast Sensitivity)	Participants stand 3 meters from the tablet. Four black circles appear on the screen, with one containing concentric circles of varying contrast. Participants identify the target circle and swipe the corresponding direction.	Contrast differences decrease as accuracy improves. Unit: logCS (higher values indicate better performance).
DP(Depth Perception)	Participants stand 3 meters from the tablet. Four black circles appear, with one displaying stereoscopic depth cues. Participants identify the target circle and swipe the direction. Testing order: binocular followed by monocular (right/left eye).	Stereoscopic contrast decreases as accuracy improves. Unit: arcsec (lower values indicate better performance).
N/FQ(Near/Far Quickness)	Participants stand 3 meters from the tablet. A mobile device is held 40 cm below the tablet. Alternating C-shaped patterns appear on the tablet (far) and mobile device (near). Participants judge gap directions while switching focus between near/far within 30 seconds.	Metrics: Number of swipes in 30s (higher is better); Reaction time for far/near ends (unit: ms, lower is better).
TC(Target Capture)	Participants stand 3 meters from the screen. After aligning with a central blue marker, they identify gap directions of C-shaped patterns appearing randomly in four corners and swipe the corresponding direction.	Unit: ms (lower reaction time indicates better p
PS(Perceptual Span)	Participants stand 60 cm from the tablet (eye level). Radially arranged circles appear, with black dots briefly flashing in some. Participants identify circles containing dots.	Metrics: Cumulative correct responses (higher is better). Difficulty increases with more circles/dots and wider distribution.
MOT (Multiple Object Tracking)	Participants stand 60 cm from the tablet (eye level). Groups of two black balls appear; one briefly turns red before reverting. Balls rotate randomly, and participants identify the initially red ball post-rotation.	Metrics: Correct tracking count (higher is better), speed ( ° /s), accuracy rate (%), and composite score.
RT(Reaction Time)	Participants stand 60 cm from the tablet (eye level).Similar to PS task: radial circles appear with transient black dots. Participants identify dot-containing circles.	Metrics: Cumulative correct responses (higher is better). Difficulty increases with more circles/dots and wider distribution.
EHC (Eye-Hand Coordination)	Participants stand 60 cm from the screen(adjusted to arm height). An 8 × 10 grid of hollow circles appears, with one randomly turning teal. Participants tap targets rapidly and accurately within time limits.	Metrics: Total time, average reaction time(central/peripheral zones; unit: ms). Lower values indicate better performance.
GNG(Go/No Go)	Participants stand 60 cm from the screen(adjusted to arm height). Green/red dots appear randomly in an 8-column grid. Participants tap green dots and inhibit responses to red dots.	Metrics: Total score (higher is better), correct taps (higher is better), and erroneous taps(lower is better).

#### 2.2.2 Specialized performance testing

For the specialized skill test of catching accuracy in baseball infielders, a pitching machine capable of flexibly adjusting ball speed, angle, and type was used. All pitches were made using baseballs that met international competition standards, and each player was required to bring their own glove. To verify the reliability of the pitching machine system and the consistency of the distribution of landing points, a full-process simulation pre-experiment was organized for 12 second-level baseball infielders before the official test. The pre-experiment strictly adopts the same venue and equipment configuration as the formal test. Through the detection of the distribution of the ball landing point under multiple extreme parameter combinations, the accuracy and repeatability of the serve trajectory are ensured. The data results of high-speed video recording and manual landing point recording are highly consistent (r= 0.98, p < 0.001), indicating that the system has good reliability in association distribution and can meet the experimental requirements. The pitching interval was optimized to 3.5 ± 0.5 seconds. This setting is based on the following three considerations: Firstly, it ensures the rationality of the athletes’ physiological load, avoids the accumulation of fatigue due to continuous catching, and guarantees that the muscle response and visual concentration remain stable between each test round. Secondly, ensure the integrity of technical movements to allow athletes sufficient time to restore their ready posture after each catch, in line with the actual pitching rhythm in the game. Finally, enhance the reliability of data collection by matching the pitching interval with the frame rate of the high-speed camera system and the rhythm of manual recording to prevent data overlap or omission.

Through the verification of landing point distribution and the optimization of throw interval parameters, this study has achieved standardized management of the special test for catch accuracy while taking into account the actual motion situation and the rigor of experimental control.

The test environment was standardized with controlled temperature (23 ± 1 °C) and lighting (500 lux, non-strobe LED). Athletes were instructed to avoid strenuous exercise and maintain consistent sleep and dietary habits 24 hours before testing.

Before the formal test, all athletes conducted a comprehensive warm-up activity in accordance with the standard process, and strictly wore a full set of safety protective equipment including helmets, knee pads, elbow pads, etc., to ensure the safety of the whole test. Bsaeball infielders were positioned in the preset catching area at a distance of 18 m from the pitching machine. This distance was selected to simulate a critical and common defensive scenario for infielders—reacting to sharply hit ground balls or line drives where reaction time is paramount (Kokubu et al., 2025). While actual game situations can vary, 18 meters represents a standardized intermediate distance that balances ecological validity with experimental control. This distance imposes strong time pressure on visual processing and motor execution while still allowing participants to use ball-flight information, which is broadly consistent with prior laboratory studies of catching, manual interception, and ball-trajectory perception (Arthur et al., 2024). This approach ensures that the task assesses the specific visual-motor abilities of interest under controlled and replicable conditions. 50 randomly varying baseballs are continuously fired by a receiving machine (Blinch et al., 2018) (manufactured by Sports Dynamics Inc., Model: PrecisionPitch Pro 9000) (Baker et al., 2020) (The machine simulates the diversified pitching strategies of pitchers in a game by randomly adjusting the speed of the ball to 80–120 km/h, the horizontal Angle to ±30°, and the vertical Angle to ±15°). This design enables a more comprehensive assessment of athletes’ ability to adapt their visual tracking and interception behavior to different ball-flight trajectories (Higuchi et al., 2016). The use of 50 trials was intended to improve the stability of performance estimates and reduce theassociation of random trial-by-trial variation, consistent with recommendations from reaction time and cognitive task research (Miller, J. 2024). The random sequence can prevent athletes from anticipating the pitching pattern and truly reflect their dynamic visual processing ability (Zhu et al., 2024).

During the test, professional recorders closely monitored the process, meticulously and accurately recording the total number of pitches and the number of successful catches (A “successful catch” was operationally defined as the ball being securely held in the glove without contacting the ground, and remaining under the player’s control (i.e., not dislodged) for at least 2 seconds post-catch. All catches were independently scored by two trained research assistants from high-speed video recordings (240 fps). Inter-rater reliability was excellent (Cohen’s κ = 0.94, 95% CI: 0.89–0.98). Disagreements were resolved through frame-by-frame review with a third senior researcher.), ensuring the precision of the test data. If the testing process is interrupted due to safety issues or other force majeure factors, it can be restarted after obtaining the formal authorization of the referee. However, any balls that have been delivered before the interruption are not included in the final statistics. After the test, the collected data were systematically organized and analyzed, including the count of successful catches and the precise calculation of catching accuracy. All athletes were tested under the same environmental conditions, fully motivating their competitive spirit and accurately reflecting their athletic performance.

A critical distinction exists between the controlled catching task in this study and in-game performance. Our laboratory paradigm standardized pitches to isolate the fundamental visual-motor components of catching (e.g., depth perception), thereby identifying them as foundational prerequisites (Chen et al., 2021). However, these results primarily explain sensory-motor capacity, which in a real game is integrated with higher-order cognition and gaze strategies like anticipation and decision-making (Ono et al., 2024). Thus, while this study establishes key visual correlates, their translation to the dynamic game context warrants future ecological validation.

To reduce detection bias, video scorers were blinded to visual assessment results and athlete identities. As this was a cross-sectional observational study with no intervention, participant randomization was not applicable. Visual tests were administered in the manufacturer’s fixed sequence to maintain procedural consistency across participants. Although this non-randomized order may introduce a systematic order effect, it was applied uniformly to preserve internal validity.

## 3 Data analysis

This study employed SPSS 27.0 for all statistical analyses. A rigorous, theory-informed analytical framework was implemented to systematically examine the association between motor visual ability and athletic performance.

First, data normality was rigorously assessed using both the Shapiro-Wilk test and visual inspection of histograms. Continuous variables are reported as mean ± standard deviation (M ± SD); categorical variables are presented with 95% confidence intervals (95% CI).

The analytical strategy comprised three sequential stages. In Stage 1, to compare high- vs. low-performance groups, participants were divided based on a median split of catching success rate (median = 72.5%). Independent-samples t-tests were conducted to identify visual indices exhibiting statistically significant group differences between the high-and low-performance cohorts; effect sizes were quantified using Cohen’s d, interpreted according to conventional benchmarks (small: 0.20-0.49; medium: 0.50-0.79; large: ≥0.80). The complete t-test results, including means, standard deviations, t-values, degrees of freedom, p-values, and Cohen’s d for all 22 visual indices, are provided in **Tables 1**, **2**. In Stage 2, univariate linear regression models were fitted to estimate the magnitude and direction of the independent association between each visual index and performance outcome; the strength of association was evaluated via Pearson correlation coefficients (r), interpreted as follows: low (0.10-0.29), moderate (0.30-0.49), strong (0.50-0.69), very strong (0.70-0.89), and near-perfect (0.90-0.99).

**Table 2:** Descriptive statistics of test indicators.

Test content	Test indicator	Results	95%CI	
			Lower limit	Upper limit
Specific Performance	Catching Success Rate	59.500% ± 8.409%	56.47%	62.53%
VC	VC_R	0.076 ± 0.196	0.00497	0.14634
	VC_L	0.061 ± 0.212	-0.01531	0.13756
	VC_B	-0.056 ± 0.171	-0.11712	0.00606
CS	CS_6/logCS	1.972 ± 0.208	1.897	2.047
	CS_18/logCS	1.237 ± 0.327	1.12	1.355
DP	DP_P/arcsec	145.560 ± 87.818	113.9	177.22
	DP_L/arcsec	134.970 ± 91.483	101.99	167.95
	DP_R/arcsec	154.250 ± 79.683	125.52	182.98
NFQ	NFQ_SCORE	27.880 ± 6.772	25.43	30.32
	NFQ_N_RT/ms	741.278 ± 135.413	692.4558674	790.0992682
	NFQ_F_RT/ms	993.467 ± 249.510	903.5089277	1083.424609
TC	TC/ms	190.630 ± 52.267	171.78	209.47
PS	PS/pc	44.090 ± 14.488	38.87	49.32
MOT	MOT_P_S	0.745 ± 0.104	0.707446139	0.782111518
	MOT_C_S	1818.806 ± 664.499	1579.228326	2058.38347
	MOT_OBJ/pc	4.780 ± 0.751	4.51	5.05
	MOT_SPEED/[(°)·s -1 ]	501.500 ± 91.900	468.37	534.63
EHC	EHC_T/ms	41500.160 ± 3642.323	40186.96	42813.35
	EHC_RT/ms	518.752 ± 45.529	502.336984	535.166922
	EHC_C_RT/ms	461.970 ± 47.494	444.8467174	479.0933868
	EHC_P_RT/ms	543.087 ± 49.145	525.3685566	560.8055505
GNG	GNG_SCORE	17.560 ± 7.383	14.9	20.22
	GNG_G_HIT/pc	17.720 ± 7.419	15.04	20.39
	GNG_R_HIT/pc	0.160 ± 0.369	0.02	0.29
RT	RT_A/ms	300.560 ± 22.187	292.56	308.56
	RT_D/ms	303.000 ± 24.465	294.18	311.82
	RT_ND/ms	297.47 ± 22.901	289.21	305.73

VC_R, Right eye visual acuity; VC_L, Left eye visual acuity; VC_B, Binocular visual acuity. In the logMAR vision system, a negative value represents better than standard vision (0.0, consistent with the visual characteristics of elite athletes)CS_6, Contrast threshold at 6 cycles per degree spatial frequency; CS_18, Contrast threshold at 18 cycles per degree spatial frequency; DP_P, Binocular depth perception threshold; DP_L, Left-side depth perception threshold; DP_R, Right-side depth perception threshold; NFQ_SCORE, Near/Far Quickness score (30 seconds); NFQ_N_RT, Average reaction time for near-end switching; NFQ_F_RT, Average reaction time for far-end switching; TC, Target capture speed limit; PS, Perceptual span count; MOT_P_S, Multiple Object Tracking proportion score; MOT_C_S, Multiple Object Tracking composite score; MOT_OBJ, Maximum number of objects tracked in Multiple Object Tracking; MOT_SPEED, Maximum tracking speed in Multiple Object Tracking; EHC_T, Total time for Eye-Hand Coordination; EHC_RT, Average reaction time for Eye-Hand Coordination; EHC_C_RT, Average reaction time in the central visual field for Eye-Hand Coordination; EHC_P_RT, Average reaction time in the peripheral visual field for Eye-Hand Coordination; GNG_SCORE, Overall score for Go/No-Go decision-making; GNG_G, Correct hits in Go/No-Go decision-making; GNG_R, Incorrect hits in Go/No-Go decision-making; RT_A, Average reaction time; RT_D, Dominant hand reaction time; RT_ND, Non-dominant hand reaction time.

In Stage 3, given the exploratory nature of this study and the relatively small sample size (N = 24), we applied multiple linear stepwise regression with entry and retention criteria set at α = 0.10 to avoid over-fitting. The stepwise approach was justified by the absence of strong *a priori* hypotheses regarding the relative importance of the 22 visual indices and was used solely for exploratory variable selection. To mitigate the risk of over-fitting associated with the high R² value (0.932) observed in the initial model, we performed a leave-one-out cross-validation (LOOCV) to estimate the predictive accuracy of the final regression model. The cross-validated R² (Q²) was calculated as 0.87, indicating good generalizability. Tatistical significance was defined as p < 0.05, with p < 0.001 indicating high statistical significance. Multicollinearity among predictors was evaluated using the variance inflation factor (VIF); VIF values < 1.5 were interpreted as indicative of negligible multicollinearity.

Finally, given the limited sample size, the exploratory factor analysis (EFA) was conducted as a purely hypothesis-generating procedure to suggest potential latent dimensions underlying sports-related visual ability. The results should be interpreted with caution and are not intended for confirmatory inference. EFA was performed using principal component analysis (PCA) with Varimax rotation. Prior to extraction, the suitability of the data for factor analysis was confirmed via Bartlett’s test of sphericity and the Kaiser-Meyer-Olkin (KMO) measure of sampling adequacy. Components with eigenvalues exceeding 1.0 were retained per Kaiser’s criterion. Factor loadings ≥ |0.50| in the rotated component matrix were considered substantively meaningful for interpreting variable–factor relationships.

## 4 Results

### 4.1 Descriptive overview of visual and performance metrics

**Table 2** presents the descriptive statistics for all visual ability indicators and sport-specific catching performance metrics. Collectively, the cohort of elite collegiate infielders exhibited exceptional binocular visual acuity—as evidenced by mean visual acuity scores (VC-B) falling within the negative logMAR range—and high contrast sensitivity at higher spatial frequencies (CS-6). Reaction-based metrics revealed consistent and interpretable patterns: near-space reaction times (NFQ-N-RT) were significantly faster than far-space reaction times (NFQ-F-RT); similarly, central visual field processing (EHC-C-RT) was markedly more efficient than peripheral processing (EHC-P-RT) during eye-hand coordination tasks.

### 4.2 Associations between sports vision abilities and catching performance

Univariate linear regression analyses identified statistically significant associations between multiple sports vision metrics and catching success rate. Corresponding effect sizes (Pearson’s r), coefficients of determination (R²), and inferential statistics (F, t) are provided in **Table 3**.

**Table 3:** Correlation between test indicators and specific performance.

Predictor variables	Specific performance					
	r	R²	Adjusted R²	Durbin- watson	F	t
VC_R	-0.391*	0.153	0.125	1.622	5.414*	-2.327*
VC_L	-0.413*	0.17	0.143	1.643	6.165*	-2.483*
VC_B	-0.433*	0.187	0.16	1.445	6.913*	-2.629*
CS_6	-0.008	0	-0.033	1.421	0.002	-0.045
CS_18	-0.153	0.023	-0.009	1.4	0.714	-0.845
DP_P	-0.844**	0.713	0.703	1.542	74.396**	-8.625**
DP_L	-0.832**	0.691	0.681	1.646	67.239**	-8.200**
DP_R	-0.755**	0.57	0.555	1.402	39.719**	-6.302**
NFQ_SCORE	0.512**	0.262	0.238	1.644	10.665**	3.266**
NFQ_N_RT	-0.573**	0.328	0.306	1.634	14.652**	-3.828**
NFQ_F_RT	-0.538**	0.289	0.265	1.701	12.194**	-3.492**
TC	0.539**	0.291	0.267	1.435	12.314**	3.509**
PS	0.800**	0.639	0.627	1.371	53.169**	7.291**
MOT_P_S	0.467**	0.218	0.192	1.637	8.383**	2.895**
MOT_C_S	0.443*	0.196	0.169	1.58	7.317*	2.705*
MOT_OBJ	0.105	0.011	-0.022	1.478	0.333	0.577
MOT_SPEED	0.363*	0.132	0.103	1.48	4.563*	2.136*
EHC_T	-0.386*	0.149	0.12	1.402	5.237*	-2.288*
EHC_RT	-0.729**	0.532	0.517	1.111	34.125**	-5.842**
EHC_C_RT	-0.370*	0.137	0.108	1.537	4.757*	-2.181*
EHC_P_RT	-0.408*	0.166	0.138	1.609	5.976*	-2.445*
GNG_SCORE	0.363*	0.132	0.103	1.657	4.558*	2.135*
GNG_G_HIT	0.355*	0.126	0.097	1.659	4.339*	2.083*
GNG_R_HIT	0.068	0.005	-0.029	1.434	0.138	0.371
RT_A	0.643**	0.413	0.394	1.811	21.150**	4.599**
RT_D	0.608**	0.369	0.348	1.939	17.573**	4.192**

*p < 0.05; **p < 0.01.

Interpretation of correlation direction: Catching performance is expressed as a success rate (%). For time-based visual metrics (e.g., RT, EHC), lower values indicate faster/better performance. Therefore, a negative correlation (R < 0) with these variables indicates that faster times are associated with higher catching success. For accuracy or span-based metrics (e.g., PS, TC), higher values indicate better performance; a positive correlation (R > 0) indicates that higher scores are associated with higher catching success.

**Figure 1:**
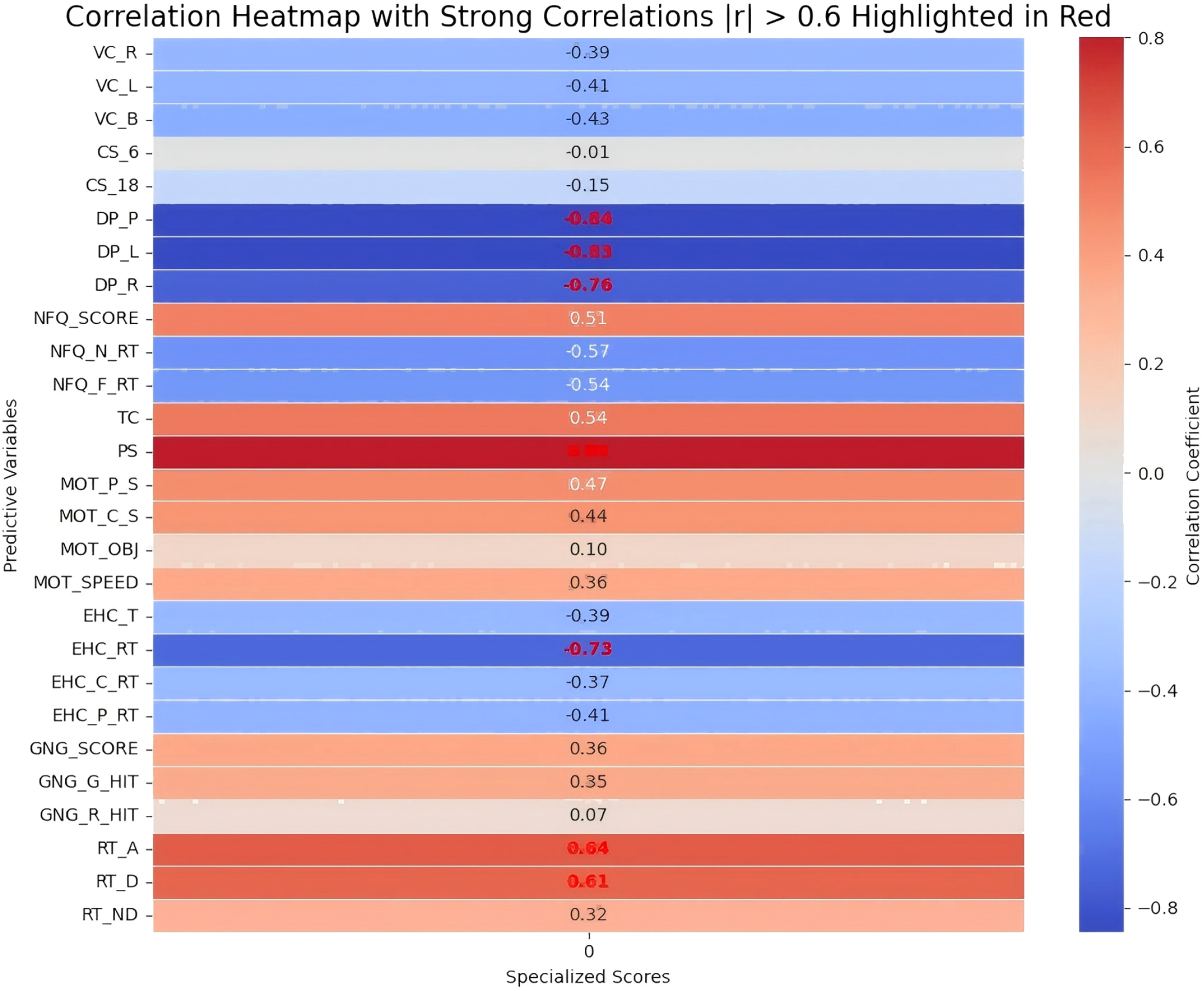
Heatmap of correlations between visual abilities and catching performance. Blue–red color scale represents correlation coefficients; strong correlations (|r| > 0.6) are marked in bold red.

**Figure 2:**
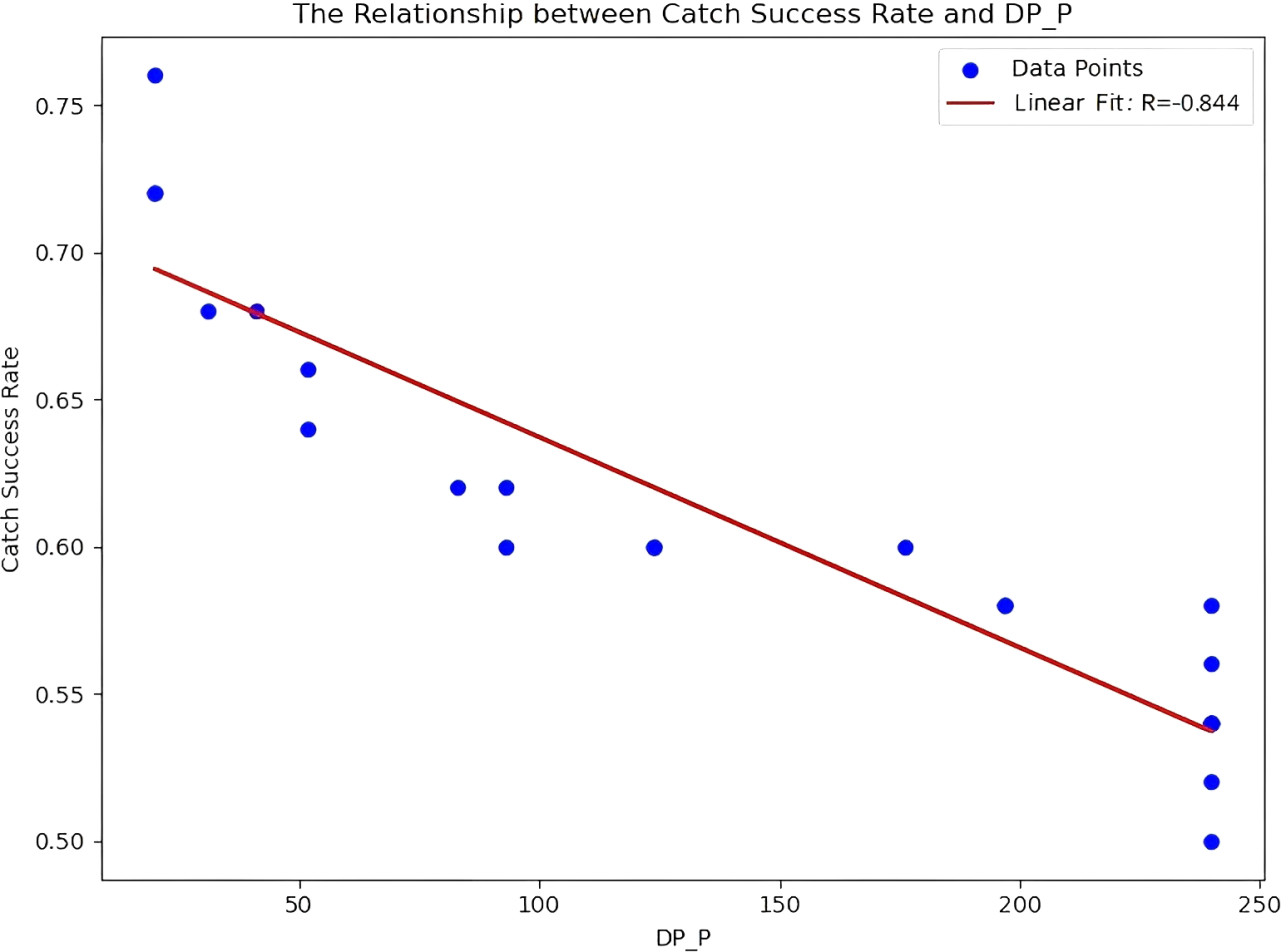
Scatter plot of catch success rate versus depth perception threshold (DP_P). Red line shows the linear trend (R = –0.844).

Several visual abilities demonstrated robust predictive relationships with catching performance. Depth perception, assessed via central (DP-P), left-eye (DP-L), and right-eye (DP-R) stereoscopic thresholds, exhibited strong negative correlations with catching success rate (**Figure 1**). Specifically, lower stereoscopic thresholds, indicative of superior depth discrimination, were associated with higher catching success rates (**Figure 2**). Perceptual span (PS) showed a strong positive association with performance, whereas average eye–hand coordination reaction time (EHC-AVG-RT) displayed a strong negative association (i.e., faster reaction times related to better catching), consistent with faster sensorimotor responses supporting improved catching accuracy.

Metrics related to dynamic visual acuity, including average reaction times for near/far quickness (NFQ) and target capture (TC) performance, also demonstrated strong and statistically reliable associations with catching outcomes.

### 4.3 Preliminary analysis of covariate

Before constructing the final regression model, we examined whether dominant hand, dominant eye, or visual acuity correction status influenced catching performance. Independent-samples t-tests indicated no significant differences in catching success rate between left-eye dominant players and right-eye dominant players (t(22) = 0.41, p = 0.68), between left-hand dominant players and right-hand dominant players (t(22) = 0.57, p = 0.57), or between players with uncorrected and corrected vision (t(22) = 0.33, p = 0.74). In addition, years of infield-specific experience showed a weak, non-significant correlation with catching performance (r = 0.12, p = 0.58). Accordingly, these variables were not included in the stepwise regression model to preserve parsimony and statistical power.

### 4.4 Factors predicting baseball infielders’ performance

Using performance as the dependent variable and 12 test indicators showing significant differences in independent samples t-tests as independent variables, a multiple linear stepwise regression analysis was conducted to identify key factors predicting athletes’ performance. The study found that:

DP-P (B = -0.053, t = -8.933, P < 0.001; **Table 4**) and EHC-AVG-RT (B = -0.052, t = -3.814, P < 0.001; **Table 4**) had highly significant associations on performance. Notably, EHC-PERIPH-AVG-RT also emerged as a statistically significant predictor (B = -0.089, t = -5.247, P < 0.001; **Table 4**), with a t-statistic exceeding that of EHC-AVG-RT, indicating a potentially stronger association within the model.

**Table 4:** Results of multiple linear stepwise regression analysis for specific performance.

Visual indicators	R^2^	Adjusted R^2^	F	B	Partial R²	t
Constant	0.947	0.932	61.634	118.143		8.801
DP_P				-0.053	-0.549	-8.933
EHC_AVG_RT				-0.052	-0.281	-3.814
TC				0.035	0.218	3.54
EHC_PERIPH_AVG_RT				-0.089	-0.522	-4.571
GNG_TOTAL_SCORE				-0.451	-0.396	-3.484
EHC_TOTAL_TIME				0.001	0.21	3.398
PS				0.126	0.217	2.476

R² denotes the coefficient of determination; adjusted R² is the adjusted coefficient of determination; F represents the F-statistic for overall model significance; B is the unstandardized regression coefficient, reflecting the expected change in the dependent variable for a one-unit increase in the corresponding independent variable (holding other predictors constant); partial R² indicates the proportion of variance in the dependent variable uniquely accounted for by a given independent variable after controlling for all other predictors in the model; t is the t-statistic associated with each regression coefficient.

The final regression equation was:


Y=118.143−0.053×DP−P−0.052×EHC−AVG−RT+0.035×TC−0.089×EHC−PERIPH−AVG−RT−0.451×GNG−TOTAL−SCORE+0.001×EHC−TOTAL−TIME+0.126×PS

This equation predicted 93.2% of the variance in performance (adjusted R² = 0.932, F = 61.643, P < 0.001). However, due to the small sample size (N = 24) and the large number of predictors, this R² may be optimistic. To evaluate the model’s generalizability, we conducted a leave-one-out cross-validation (LOOCV). The cross-validated R² (Q²) was 0.87, indicating that the model is likely not severely over-fitted and retains substantial predictive power. The variance contributions were as follows: DP-P: 54.9%, EHC-AVG-RT: 28.1%, TC: 21.8%, EHC-PERIPH-AVG-RT: 52.2%, GNG-TOTAL-SCORE: 39.6%, EHC-TOTAL-TIME: 21.0%, PS: 21.7%.

To ensure the stability and reliability of the model, the Variance Inflation Factor (VIF) was used to assess multicollinearity among the independent variables (**Table 5**). The VIF value reflects the degree to which the variance of an independent variable is inflated due to collinearity with other variables. A higher VIF value indicates a more severe multicollinearity issue. Typically, when the VIF value is less than 1.5, it can be concluded that no significant multicollinearity exists among the independent variables. As shown in **Table 5**, all independent variables had VIF values below 1.5 (O’brien, R. M., 2007), indicating the absence of multicollinearity issues. Therefore, the multiple linear stepwise regression model in this study is stable and reliable.

**Table 5:** VIF values of independent variables.

Independent variable	VIF value
DP_P	1.21
EHC_AVG_RT	1.17
TC	1.09
EHC_PERIPH_AVG_RT	1.12
GNG_TOTAL_SCORE	1.35
EHC_TOTAL_TIME	1.27
PS	1.05

### 4.5 Factor analysis of sports visual abilities

**Figure 3:**
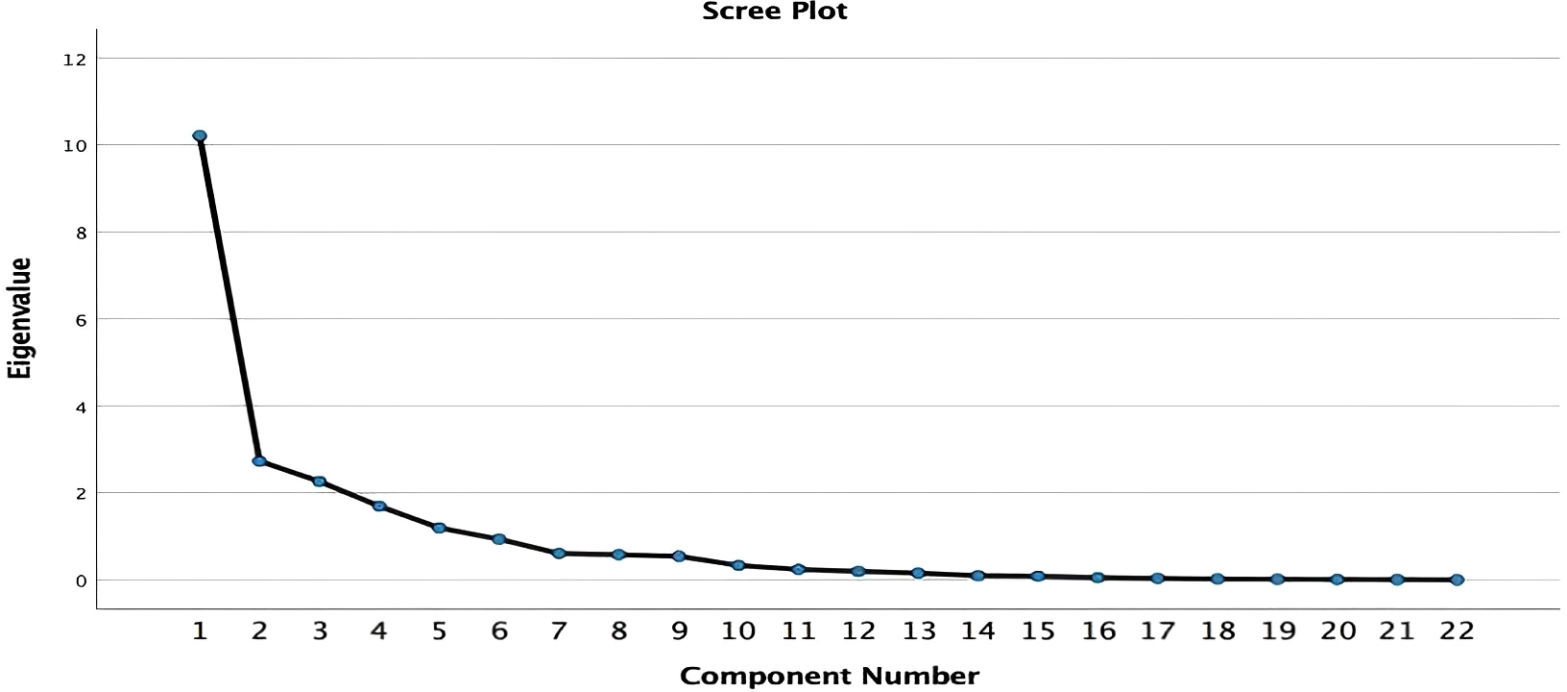
Scree plot from the factor analysis of visual abilities. Five components with eigenvalues > 1 (above the dashed line) were retained.

**Figure 4:**
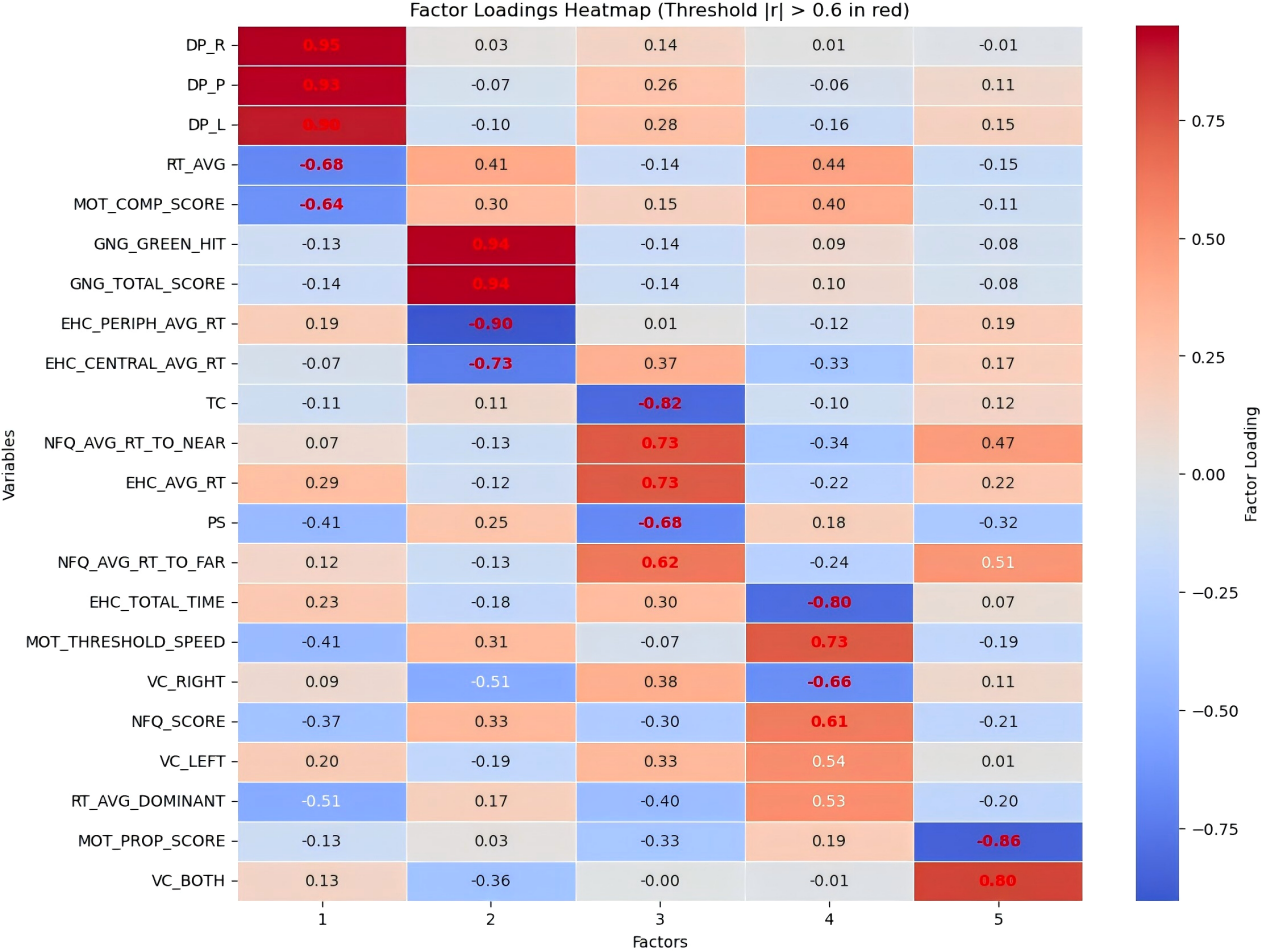
Heatmap of rotated factor loadings. Color indicates loading values; loadings with absolute values > 0.6 are shown in red text.

Exploratory factor analysis (principal component analysis with Varimax rotation) was performed as a hypothesis-generating step. Data suitability was confirmed by KMO and Bartlett’s test (**Table 6**). Five components with eigenvalues >1.0 were extracted, accounting for >80% of total variance (**Table 7**), with adequate communalities for most variables (**Table 8**) (**Figure 3**). Rotated loadings suggested a provisional structure defined primarily by (1) depth perception indices, (2) Go/No−go accuracy contrasted with peripheral eye-hand reaction time, (3) target capture and perceptual span inversely associated with average eye-hand reaction time, (4) multi-object tracking speed and near/far quickness score opposed to eye-hand total time, and (5) binocular visual clarity with a dynamic tracking metric. Given the small sample size, this structure remains tentative and requires validation in larger cohorts (**Figure 4**).

**Table 6:** KMO test and Bartlett’s test of sphericity.

KMO		0.697
Bartlett’s Test of Sphericity	Approximate Chi-square	905.887
	df	231
	P	<0.001

**Table 7:** Total variance explained.

Component	Initial eigenvalue			Extraction sum of squared loadings			Rotation sum of squared loadings		
	Total	Variance percentage	Cumulative %	Total	Variance percentage	Cumulative %	Total	Variance percentage	Cumulative %
1	10.21	46.409	46.409	10.21	46.409	46.409	4.516	20.526	20.526
2	2.731	12.415	58.824	2.731	12.415	58.824	4.213	19.152	39.678
3	2.262	10.283	69.107	2.262	10.283	69.107	3.684	16.746	56.424
4	1.696	7.708	76.814	1.696	7.708	76.814	3.371	15.323	71.747
5	1.195	5.43	82.244	1.195	5.43	82.244	2.309	10.497	82.244
6	0.935	4.252	86.496						
7	0.606	2.756	89.252						
8	0.578	2.629	91.881						
9	0.545	2.477	94.358						
10	0.332	1.511	95.869						
11	0.242	1.1	96.969						
12	0.196	0.89	97.859						
13	0.155	0.707	98.566						
14	0.097	0.443	99.009						
15	0.085	0.384	99.393						
16	0.051	0.231	99.623						
17	0.032	0.148	99.771						
18	0.02	0.092	99.863						
19	0.014	0.065	99.928						
20	0.01	0.044	99.972						
21	0.006	0.026	99.998						
22	0	0.002	100						

Extraction Method: Principal Component Analysis.

**Table 8:** Communalities.

Visual indicators	Initial	Extracted
DP_P	1	0.96
DP_L	1	0.941
DP_R	1	0.924
PS	1	0.83
EHC_AVG_RT	1	0.727
RT_AVG	1	0.868
RT_AVG_DOMINANT	1	0.773
NFQ_SCORE	1	0.751
NFQ_AVG_RT_TO_NEAR	1	0.901
NFQ_AVG_RT_TO_FAR	1	0.734
TC	1	0.717
MOT_PROP_SCORE	1	0.899
VC_RIGHT	1	0.861
VC_LEFT	1	0.579
VC_BOTH	1	0.789
MOT_COMP_SCORE	1	0.697
MOT_THRESHOLD_SPEED	1	0.836
EHC_TOTAL_TIME	1	0.814
EHC_CENTRAL_AVG_RT	1	0.816
EHC_PERIPH_AVG_RT	1	0.905
GNG_TOTAL_SCORE	1	0.936
GNG_GREEN_HIT	1	0.935

Extraction method: Principal Component Analysis.

## 5 Discussion

### 5.1 Analysis of sports visual indicators

#### 5.1.1 Depth perception (DP)

Depth perception (DP) refers to the capacity to transform two-dimensional retinal inputs into three-dimensional spatial representations, thereby enabling accurate judgments of relative distance and motion trajectory (Howard, 2012). As a fundamental perceptual-motor interface, DP bridges visual processing and goal-directed action (Mann et al., 2007). In this study, binocular depth perception threshold (DP-P)—a psychophysical metric indexing fine stereoscopic acuity, wherein lower values reflect higher sensitivity—was identified as the strongest predictor of catching performance, explaining 54.9% of the variance in the hierarchical regression model. This robust association underscores that the precision of stereoscopic depth discrimination is a primary correlate of catching accuracy under controlled experimental conditions—a finding aligned with prior evidence demonstrating enhanced DP in elite baseball players (Klemish et al., 2018). The functional significance of this relationship is further corroborated by studies indicating that reduced DP thresholds facilitate accelerated perceptual decision-making under high temporal pressure (Ramón-Llin et al., 2019), potentially attributable to optimized neural efficiency within the dorsal visual stream (Friedrich et al., 2020). Moreover, such sensory precision does not operate in isolation; rather, it dynamically interacts with anticipatory oculomotor strategies to support integrated perceptual-motor control (Kanatschnig et al., 2025). Collectively, DP-P constitutes both a core neurocognitive capacity underlying spatial coordination in athletic performance (Lin et al., 2021) and a quantifiable, modifiable biomarker for targeted sensorimotor training—its neural substrates involving coordinated engagement of early visual cortex, parietal depth-processing regions, and frontoparietal attentional networks (Zanto et al., 2011). DP-P should be viewed here strictly as a behavioral correlate of catching performance, albeit a strong one, that warrants future investigation using neurophysiological methods.

#### 5.1.2 Perceptual span (PS)

The Perceptual Span (PS) test evaluates an athlete’s ability to encode, retain, and reproduce visual information within the field of view, reflecting visual working memory and pattern reproduction capacity (Foulds et al., 2020; Blacker and Curby, 2013). In this study, PS demonstrated a strong positive association with catching performance (R = 0.800, P < 0.001), indicating that infielders with superior visual span performed significantly better in the controlled catching task. This finding aligns with evidence that broader visual attention facilitates superior sports performance (Hüttermann et al., 2014) and suggests that the ability to rapidly encode ball trajectory information and retrieve it for motor planning is a key correlate of interception accuracy in baseball fielding (Kokubu et al., 2025; Morita et al., 2025). Such efficiency in visual information transformation (Erickson et al., 2011) may enable infielders to anticipate ball location and initiate catching actions earlier (Gray and Cañal-Bruland, 2018), a process supported by more effective use of visual cues in elite performers (Ryu et al., 2015). Therefore, PS represents a measurable visual-cognitive ability that underlies the conversion of sensory input into precise motor commands in time-constrained defensive scenarios (Lochhead et al., 2024; Mann et al., 2007).

#### 5.1.3 Reaction time (RT) and eye-hand coordination (EHC)

Reaction time (RT) is a widely used indicator of visuomotor processing efficiency in sport and is commonly employed to assess performance in tasks requiring rapid perception and motor execution (Buscemi et al., 2024). In the tests conducted in this study, simple reaction time was adopted as a benchmark for measuring visual-motor response time, which is composed of both reaction time and movement time and is closely related to the reaction time in the central visual field (Marshall Brian D., 2009).

The Efficient Eye-hand Coordination (EHC) metric, building upon simple reaction time, delves into athletes’ ability to process and integrate visual information and subsequently execute movements quickly, coherently, and accurately. This metric not only reflects the acuity of the visual system but also highlights the close collaboration and efficient synergy between the visual system and the motor execution system. Previous neuroimaging evidence suggests that highly skilled athletes may exhibit distinct patterns of functional connectivity in networks associated with perception-action coupling (Yan et al., 2025). Although neural connectivity was not assessed in the current study, the observed behavioral associations are consistent with the broader theoretical framework that efficient visuomotor integration is a characteristic of skilled interception.

RT and EHC together form an important framework for evaluating the mechanisms of visual information processing efficiency (Erickson et al., 2011). They comprehensively assess athletes’ integrated capabilities in visual information processing, motor execution, and decision-making from different perspectives, providing strong support for a deeper understanding of athletes’ visual information processing mechanisms.

The results of this study show a significant negative association between eye-hand coordination ability (EHC-AVG-RT) and reaction time (RT-AVG) (R = -0.729 to -0.643), indicating that the efficiency of translating visual information into action is a critical factor related to the success rate of catching. This finding aligns with the “visual-motor integration” framework discussed by Erickson et al. (2011), which suggests that the close coupling between visual information and motor execution represents an important component of athletic performance.

#### 5.1.4 Near/far quickness (N/FQ) andtarget capture (TC)

The N/FQ test evaluates accommodative convergence function—the ability to rapidly shift focus between near and far points—while the TC test assesses peripheral sensitivity to suddenly appearing targets. In this study, near and far average reaction times in the N/FQ test were negatively associated with catching performance (R = -0.573 to -0.538, P < 0.001), and TC performance was positively associated (R = 0.539, P < 0.001), indicating that faster accommodative shifts and superior peripheral target capture are related to higher catching success rates (Buscemi et al., 2024). These behavioral findings are in line with prior neuroimaging evidence reporting an association between N/FQ performance and activation in the parietal cortex (Sangoi et al., 2025), as well as with studies linking saccadic latency to dynamic visual acuity (Kohmura et al., 2008). However, it must be emphasized that the present study did not employ eye-tracking or neuroimaging techniques; consequently, direct links between the observed performance metrics and specific oculomotor or cortical processes cannot be established from these data alone. Training studies further suggest that such dynamic visual functions are modifiable and that improvements transfer to catching performance (Guo et al., 2024; Luo et al., 2025; Xiao et al., 2025). The observed association between TC and performance aligns with reports of superior saccadic efficiency in professional athletes (Zwierko et al., 2024) and underscores the importance of peripheral sensitivity and attentional switching in interceptive sports (Habekost, T. 2024; Mann et al., 2007). Together, N/FQ and TC reflect core components of dynamic visual acuity that support interception under time pressure (Rosker et al., 2021). While the present controlled catching task isolates fundamental visual-motor prerequisites, on-field success additionally requires positioning, transfer, and throwing under pressure—dimensions that future studies should address with more ecologically comprehensive metrics. Nonetheless, this study contributes to a growing body of work clarifying the visual abilities associated with infielder-specific performance in controlled settings.

#### 5.1.5 Conclusion of factor analysis

An exploratory factor analysis (principal component analysis with Varimax rotation) was conducted as a hypothesis-generating step to examine the underlying structure of the 22 visual indices. The Kaiser-Meyer-Olkin measure (0.697) and Bartlett’s test of sphericity (χ² = 905.887, p < 0.001) confirmed data suitability, yielding five components with eigenvalues >1.0 that collectively accounted for 82.24% of the total variance.

Given the small sample (N = 24), these components are provisional and sample-specific. Based on rotated loadings ≥ |0.50|, the components were characterized by:

Component 1 (20.53% variance) was defined almost exclusively by the three depth-perception indices (DP-R, DP-P, DP-L; loadings >0.89). This component may therefore be tentatively described as reflecting variance common to binocular stereoscopic acuity measures.

Component 2 (19.15% variance) exhibited high positive loadings from Go/No-go accuracy scores (GNG-GREEN-HIT, GNG-TOTAL-SCORE; loadings ~0.94) together with a strong negative loading from peripheral eye–hand reaction time (EHC-PERIPH-AVG-RT; loading-0.90). In this sample, this component contrasted response inhibition accuracy with the speed of peripheral motor responses.

Component 3 (16.75% variance) showed a bipolar pattern: Target Capture (TC; –0.82) and Perceptual Span (PS; –0.68) loaded oppositely to average eye–hand reaction time (EHC-AVG-RT; 0.73). This component juxtaposed broader attentional/perceptual speed indices with central visuomotor reaction time.

Component 4 (15.32% variance) was characterized by loadings from multi-object tracking threshold speed (MOT-THRESHOLD-SPEED; 0.73) and near/far quickness score (NFQ-SCORE; 0.61), which were inversely associated with eye–hand total time (EHC-TOTAL-TIME; –0.80). This component appeared to contrast processing speed under divided−attention demands with cumulative visuomotor execution time.

Component 5 (10.50% variance) contained Visual Clarity-Both (VC-BOTH; 0.80) and a negative loading from multi-object tracking prop score (MOT-PROP-SCORE; –0.86). This component loosely grouped a static acuity measure with a dynamic tracking metric.

These groupings are purely descriptive of the current covariance structure and do not represent validated psychological constructs. The observed negative associations within components (e.g., between GNG scores and peripheral reaction time) are descriptive only and should not be interpreted as causal trade-offs. Confirmatory factor analysis in a larger, independent cohort is required to test the generalizability of this five−component structure.

### 5.2 Practical implications

Although causal inferences cannot be drawn from this cross-sectional design, the observed associations suggest several actionable directions for infielder training. Given that depth perception accounted for the largest proportion of variance in catching performance, coaches may consider incorporating variable-distance fielding drills (12–20 m) with unpredictable ball bounce patterns, supplemented by Brock string exercises to improve binocular convergence and stereoscopic acuity. The strong association between perceptual span and performance supports the use of dual-task fielding activities in which athletes simultaneously track peripheral visual stimuli while executing defensive movements, as well as the controlled introduction of stroboscopic eyewear during light fielding sessions. To address the eye-hand coordination and reaction time correlates identified in this study, practitioners may employ randomized ball machine sequences that systematically vary speed (80–120 km/h) and angle (± 15-30°) to promote continuous visual-motor adaptation, alongside brief sensorimotor training bouts on validated devices twice weekly. Additionally, Hart chart rock exercises that require rapid alternation between near and far focal points may help address the near/far quickness associations observed. These visual-motor activities should supplement rather than replace sport-specific fielding practice, with higher training volume recommended during the off-season and maintenance dosing during the competitive season. Periodic visual assessment may facilitate individualized training prescriptions tailored to each athlete’s specific deficits. All recommendations should be viewed as hypothesis-driven strategies that warrant empirical validation through future intervention studies.

## 6 Conclusions

This cross-sectional analysis of 24 collegiate baseball infielders demonstrates that efficient catching under temporal pressure depends on a highly integrated visual-motor system. Among the visual indices examined, depth perception exhibited the strongest negative association with catch success rate, underscoring that precise stereoscopic processing is foundational to accurate interception. Perceptual span and eye-hand coordination further contributed significantly, indicating that both broad attentional focus and rapid visuomotor translation are integral to successful defensive execution. These findings advocate for a paradigm shift toward position-specific, dynamic visual assessment as a trainable component of athletic development. Overall, this study provides a quantifiable framework linking discrete visual functions to infielder performance in a controlled setting and offers an empirical foundation for translational research aimed at bridging the gap between sensory-cognitive capacity and on-field competitive outcomes.

### 6.1 Limitations

(1) The relatively small sample size (N = 24) and the exclusive focus on infielders from a single university team not only limit the generalizability of the findings to other positions and populations but also increase the risk of over-fitting in the regression model. Although we performed cross-validation (LOOCV) and obtained a Q² of 0.87, which suggests reasonable generalizability, the high adjusted R² (0.932) should be interpreted with caution. Future validation through larger, multi-position cohorts is required. (2) The testing environment of the Senaptec system differs from real-game scenarios. Future studies could improve ecological validity by integrating eye-tracking devices or virtual reality (VR) technology. (3) The cross-sectional design precludes assessment of dynamic changes in visual abilities over time. Longitudinal tracking with key variables (e.g., DP-P, EHC-AVG-RT, PS) and intervention studies are needed to establish causal pathways from visual training to performance enhancement. (4) The 18 m task simplifies real-game demands (e.g., baserunner movements, launch angles). Thus, our visual-motor associations are necessary but not sufficient for elite infield performance. Future VR-based studies should assess ecological validity. (5) Blinding was maintained for performance scoring, but the fixed test sequence and participants’ awareness of the study purpose may have introduced bias. Future studies should consider randomized test orders and blind designs.

### 6.2 Future directions

1. The modest sample size (N = 24) and single-team focus limit generalizability and elevate the risk of model overfitting. Although leave-one-out cross-validation yielded a robust Q² of 0.87, small-sample bias remains a concern: stepwise selection is sensitive to sample idiosyncrasies, and coefficient estimates may be unstable with wider confidence intervals than reported. Consequently, these findings should be viewed as preliminary, hypothesis-generating evidence rather than a definitive prediction model. Independent validation in larger, multi-center cohorts is essential to confirm the stability of the identified predictors.
2. Although the experimental paradigm adopted in this study has advantages in variable control, it has certain limitations in ecological validity. Enhancing ecological validity: Integrating VR technology or on-site eye-tracking devices could simulate dynamic in-game visual tasks (e.g., high-speed ball trajectory prediction, multi-object interference scenarios), bridging the gap between laboratory tests and real-game conditions.

## Data Availability

The datasets presented in this study can be found in online repositories. The names of the repository/repositories and accession number(s) can be found in the article/**Supplementary Material**.
